# Evaluation of the immediate effect of acupuncture on pain, cervical range of motion and electromyographic activity of the upper trapezius muscle in patients with nonspecific neck pain: study protocol for a randomized controlled trial

**DOI:** 10.1186/s13063-015-0623-3

**Published:** 2015-03-19

**Authors:** Simone Aparecida Penimpedo Calamita, Daniela Aparecida Biasotto-Gonzalez, Nivea Cristina De Melo, Douglas Meira dos Santos, Roberta de Lassa, Fabiana Sarilho de Mendonça, Claudia Santos Oliveira, César Ferreira Amorim, Tabajara Oliveira Gonzalez, Marco Antônio Fumagalli, Cid André Fidelis Paula de Gomes, Fabiano Politti

**Affiliations:** Postgraduate Program in Rehabilitation Sciences, Universidade Nove de Julho (UNINOVE), Av Dr Adolfo Pinto, 109, Água Branca, São Paulo, SP 05001-100 Brazil; Department of Physical Therapy, Universidade Nove de Julho (UNINOVE), Av Dr Adolfo Pinto, 109, Água Branca, São Paulo, SP 05001-100 Brazil; Physical Therapy Program, Universidade Cidade de São Paulo (UNICID), Rua Cesário Galeno, 448/475, Tatuapé, São Paulo, SP 03071000 Brazil

**Keywords:** Physical therapy, Rehabilitation, Neck pain, Acupuncture, Electromyography, Pain, Upper trapezius muscle, Clinical trial

## Abstract

**Background:**

Nonspecific neck pain can cause considerable suffering, possible disability and reductions in quality of life and productivity. The aim of the proposed study is to evaluate the immediate effect of acupuncture on pain, cervical range of motion and electromyographic activity of the upper trapezius muscle in patients with nonspecific neck pain.

**Methods/Design:**

A total of 12 patients with nonspecific neck pain and 12 healthy subjects will be enrolled in a randomized, single-blind crossover study. Each subject will receive two forms of treatment in random order: a single session of traditional acupuncture (acupoints: triple energizer 5, ‘*Wai-guan*’ and large intestine 11, ‘*Qu-chi*’) and sham acupuncture. To eliminate carry-over treatment effects, a one-week wash-out period will be respected between sessions. Surface electromyography will be used to determine motor control in the upper trapezius muscle before and after treatment. The outcome measures in the group with neck pain will be a numerical pain rating scale (range: 0 (no pain) to 10 (maximum pain)), documentation of the pain area on a body chart and cervical range of motion. Comparisons before and after acupuncture treatment will demonstrate whether acupoints affect the activity of the upper trapezius muscle, pain and cervical range of motion.

**Discussion:**

The purpose of this randomized clinical trial is to evaluate the immediate effect of acupuncture on pain, cervical range of motion and electromyographic activity of the upper trapezius muscle in patients with nonspecific neck pain. Data will be published after the study is completed. The study will support the practice of evidence-based physical therapy for individuals with nonspecific neck pain.

**Trial registration:**

This trial was registered with Clinicaltrials.gov (identifier: NCT0984021) on 7 November 2013 (https://clinicaltrials.gov/ct2/show/NCT01984021).

## Background

Individuals with nonspecific neck pain (NS-NP) exhibit mechanical alterations and signs of degeneration of the cervical spine [1], causing pain that can irradiate to the upper limbs [2], increased sensitivity, fatigue and stiffness in the muscles of the neck and shoulder, and headache that irradiates to the neck [3]. Changes in muscle behavior in such patients have been demonstrated in studies involving surface electromyography (sEMG) [4-6]. According to one study, there is strong evidence that the motor control of the upper trapezius muscle is directly affected by cervical pain during isometric activities [5]. The studies cited offer preliminary support for the use of treatment methods for NS-NP that emphasize the reestablishment of function in the muscles of the neck. Massage, scapula mobilization, physical therapy, exercise, manual therapy and acupuncture have been reported to be efficient in reducing neck pain [6-10]. However, there is a lack of consensus on the best form of treatment for such patients.

Although acupuncture is accepted and recommended for the treatment of NS-NP [11-13], the arguments used for the effects encountered and the different forms of therapeutic approach hinder a clear understanding of the physiological mechanisms involved. The main limitations regard the different techniques employed, such as either systemic acupuncture [12,14] or in combination with auricular [11,15] or cranial acupuncture [16], or the direct application of needles in trigger points [14,17]. Different numbers of acupoints and forms of needle application within the same treatment technique [11,12,17], as well as variability in the number of sessions (usually ranging from five to 10) [11,12,17], further hamper the choice of this resource for the treatment of NS-NP. In the case of individuals with NS-NP, the improvement in the clinical condition after the use of acupuncture may be attributed to the inhibition of the excitability of the alpha motor neuron [18,19]. However, a thorough review of the literature revealed no studies addressing the effect of acupuncture on motor control in any of the neck muscles in patients with NS-NP.

Changes in muscle activity are generally characterized by the variation and/or reduction in the firing rate of the motor units [20,21], reflecting changes in the myoelectrical pattern of the muscle that can be measured through sEMG [21,22]. Thus, sEMG has been used to determine whether the stimulus from acupuncture has an effect on the activity of skeletal striated muscle [19,23-26]. Some studies have demonstrated a remote effect from acupuncture in the upper trapezius muscle [19,26]. However, further investigations have not found any clinical benefits relating to this discovery. The description of the relationship between the upper trapezius muscle and the acupoints denominated triple energizer 5 (TE-5; ‘*Wai-guan*’) and large intestine 11 (LI-11; ‘*Qu-chi*’) [26] allow the use of sEMG to clarify whether acupuncture truly has a neurophysiological effect on this muscle, the motor control of which is affected in individuals with NS-NP [5].

In clinical practice, the confirmation that acupoints TE-5 and LI-11 act directly on the upper trapezius muscle in patients with NS-NP could contribute toward: i) the standardization of these acupoints as stimuli for the treatment of changes in the upper trapezius muscle stemming from mechanical injury, myofascial tension with and without the presence of trigger points and systemic diseases, such as fibromyalgia; ii) improving the estimation of the effects of treatment; iii) making the use of acupuncture less complex; iv) stimulating the use of acupuncture alone or in combination with other forms of treatment (physical therapy, exercise, massage or manual therapy) and v) allowing the testing of other noninvasive stimuli, such as low-level laser therapy.

The objectives of this study are:

1. To determine whether stimulation with acupuncture at acupoints TE-5 and LI-11 affects sEMG activity of the upper trapezius muscle in healthy subjects and patients with NS-NP and
2. To determine whether stimulation with acupuncture at acupoints TE-5 and LI-11 contributes to improvements in pain and cervical range of motion in patients with NS-NP.

The first hypothesis is that stimulation with acupuncture at acupoints TE-5 and LI-11 acts directly on sEMG activity of the upper trapezius muscle in healthy subjects and individuals with NS-NP. The second hypothesis is that stimulation with acupuncture at acupoints TE-5 and LI-11 provides immediate improvements in pain and cervical range of motion in patients with NS-NP.

## Methods/Design

### Overview of research design

**Figure 1:**
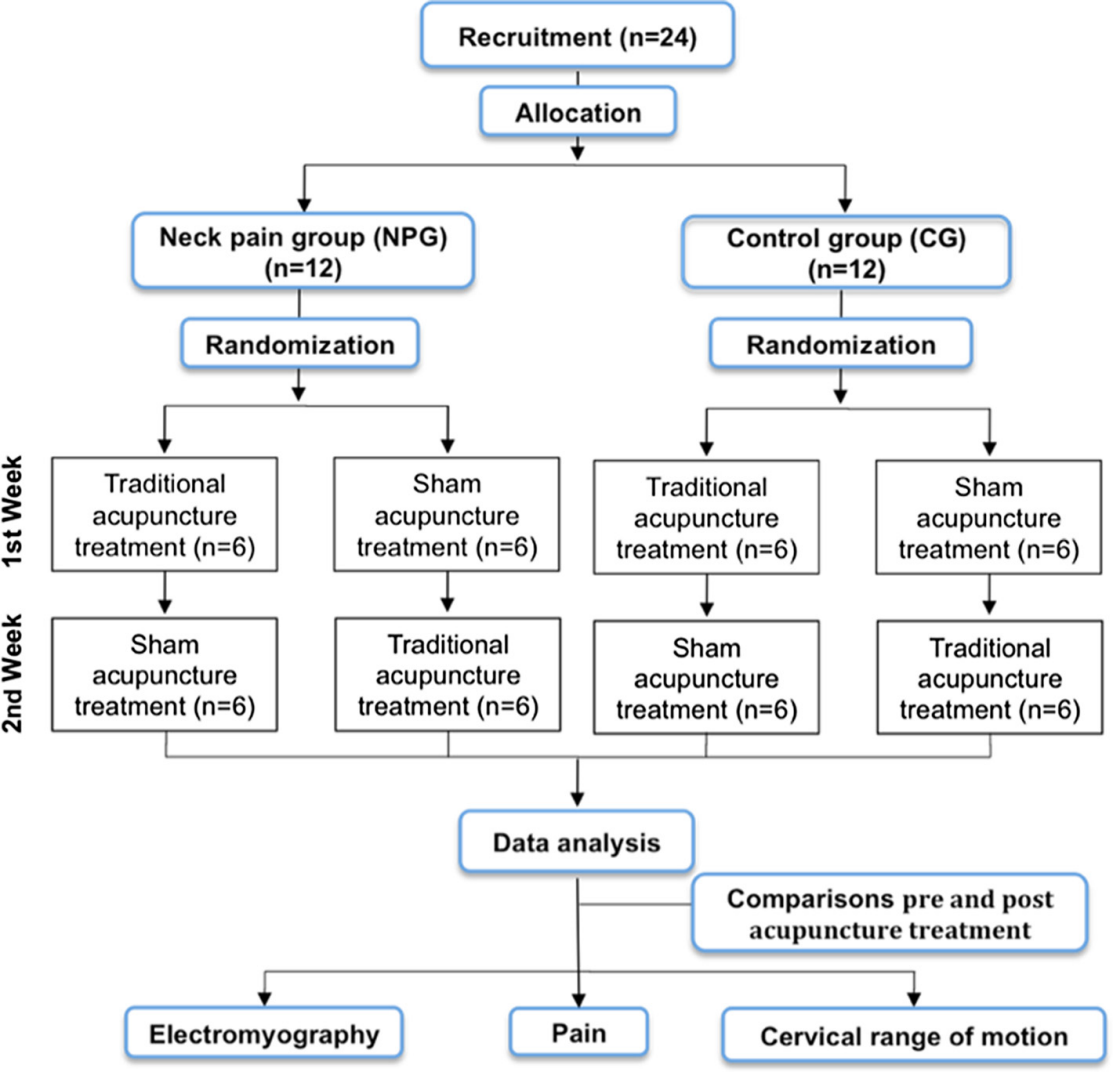
**Flowchart of the study.**

A randomized, single-blind, sham-controlled, crossover clinical trial will be carried out. The crossover design will be used to exclude the potential interference of individual differences. Figure 1 displays the flow of the study.

### Study setting

Potential participants will be recruited from patients sent for the treatment of neck pain at the rehabilitation department of the University Nove de Julho. Undergraduate students from the same university will also be asked to participate in the study as the control group (CG).

### Inclusion criteria

Individuals within an age range of 18 to 40 years will be accepted for both groups to minimize any confounding effects stemming from advanced degenerative alterations in the cervical spine. The following will be the inclusion criteria for the NS-NP group (neck pain group (NPG)): score of 15 or more on the Neck Disability Index (adapted and validated for the Brazilian population), which specifically evaluates neck pain and disability [27]; history of neck pain for a period of more than three months; restricted active or passive neck movement in at least one direction and score range of three to six points on an 11-point (range: 0 to 10) numerical rating scale for perceived pain intensity. The inclusion criteria for the CG will be no self-reported history of neck pain and no positive signs of cervical spine or scapular dysfunction during the physical examination.

### Exclusion criteria

The following will be the exclusion criteria for both groups: history of neurological disorders (irradiated pain) or neck surgery; chronic neck pain resulting from a traumatic incident; chronic musculoskeletal condition (such as muscular disorder or polyarthritis); medical diagnosis of fibromyalgia; systemic disease; connective tissue disorder; body mass index <25 kg/m; non-tolerance of needles; current pregnancy; having undergone physical therapy, massage or acupuncture in the previous two weeks or use of analgesic, muscle relaxant, psychotropic agent or anti-inflammatory agent in the previous three days.

### Randomization and blinding

Each patient will receive two forms of acupuncture in random order: a single session of traditional acupuncture (tACP) and sham acupuncture (sACP). To eliminate carry-over treatment effects, a one-week wash-out period between the treatments will be respected. Randomization in each group will be performed by lots using opaque envelopes containing either the letter A, corresponding to tACP, or B, corresponding to sACP. The participants in each group will be distributed in two subgroups of six individuals based on the initial treatment (tACP and sACP), as shown in Figure 1. When one of the subgroups has completed the treatment course, the order of consecutive arrival of the volunteers will be used until all individuals have been allocated.

Measurements will be performed by five independent researchers. Researcher one will be in charge of the screening process, administration of the questionnaire and randomization of the treatments. Researcher two will be in charge of collecting the EMG data. Researcher three will administer the acupuncture treatment. Researcher four will be in charge of the analysis and processing of the EMG data. Researcher five will be in charge of the statistical analysis of the EMG, pain and cervical range of motion data. The participants will be blinded to the type of treatment and will not be informed that one of the two treatments is a sham procedure. Researchers four and five will also be blinded to both the group allocation and type of treatment.

### Outcome measures

The primary outcome of the study is the EMG activity of the upper trapezius muscle after a single session of acupuncture. The secondary outcomes for the study are the numerical rating scale score for pain, pain area and cervical range of motion.

### Measures

#### Numerical rating scale for pain

A numerical rating scale for pain will be used to assess pain intensity in the NPG. This is an 11-point scale ranging from 0 (absence of pain) to 10 (worst possible pain) [28]. The numerical rating scale has been translated and cross-culturally adapted for the Brazilian population [29].

#### Pain area

The individuals in the NPG will be asked to draw the distribution of their overall pain symptoms on an anatomical body map. The drawings will be subsequently digitized using the ACECAD D9000 (ACE CAD Enterprice Co Ltd, Taipei Hsien, Taiwan) and pain areas will be measured.

#### Cervical range of motion

A range of motion instrument will be used to determine cervical spine movements (Model: CROM Basic, Performance Attainment Associates, Roseville, Minnesota, United States). This instrument is composed of two gravity goniometers and a compass goniometer, and has been demonstrated to be a reliable tool, with adequate validity [30]. The device will be placed on the top of the head and the patient will be instructed to move the head as far as possible, without experiencing pain, in a standard fashion: right rotation, left rotation, flexion, extension, right lateral flexion and left lateral flexion. Three trials will be conducted in randomized order for each direction of movement and mean values will be recorded for analysis.

#### Electromyography

The sEMG signal will be recorded in the dominant upper trapezius muscle in the CG and on the side with the greatest self-reported pain in the NPG. EMG signals will be obtained using an eight-channel module (Model: EMG830C, EMG System do Brazil Ltda™, São José dos Campos, São Paulo, Brasil ), with a band pass filter with cut-off frequencies of 20 to 500 Hz, an amplifier gain of 1,000 and a common rejection mode ratio of less than 120 dB. All data will be acquired and processed using a 16-bit analog-to-digital converter, with a sampling frequency of 2 kHz.

Bipolar surface circular electrodes (Ag/AgCl, Medi-Trace™, Minneapolis, Minessota, United States) with a 10 mm diameter, will be used for the surface recording of EMG, with a center-to-center distance of 20 mm. Before electrode placement, the skin will be cleaned using abrasive paste (EASYCAP™, Woerthsee-Etterschlag, Germany). The electrode will be positioned 2 cm lateral to the midpoint of the line between the C7 spinous process and the acromion [31].

### Acupuncture

In the NPG, the upper trapezius muscle with the greatest area of pain will be chosen for acupuncture. Sterile acupuncture needles measuring 0.25 × 13 mm (Suzhou Huanqiu Acupuncture Medical Appliance Co. Ltd.™, Suzhou, Jiangsy, China) will be inserted in TE-5 (located on the dorsal face of the forearm between the radius and ulna, 3 cm above the joint line of the wrist) and LI-11 (located at the outermost point of the skinfold of elbow flexion, in the direction of the lateral epicondyle of the elbow) [26]. In the CG, the needles will be inserted 1 cm to the side of TE-5 and 1 cm to the side of LI-11 (in the direction of the styloid process of the radius).

Needle insertion will be performed by a physiotherapist with 10 years of experience who is certified in the use of acupuncture. The following insertion procedures will be carried out: i) participant seated comfortably with the upper limb unclothed; ii) cleaning of the skin with alcohol at the points to receive the needles; iii) needle depth in the NPG will be until the participant feels a sensation of mild shock (denominated *de-qi*) and needle depth in the CG will be superficial (just enough to maintain the needles fixed during the experiments and not enough to produce *de-qi*) and iv) in both groups, the needles will be rotated in both the clockwise and counter-clockwise directions for 10 seconds, every three minutes, at an approximate frequency of 60 times a minute. After the experiment, all participants in the NPG will be sent for physical therapy at the rehabilitation clinic of the University Nove de Julho.

### Experimental procedures

**Figure 2:**
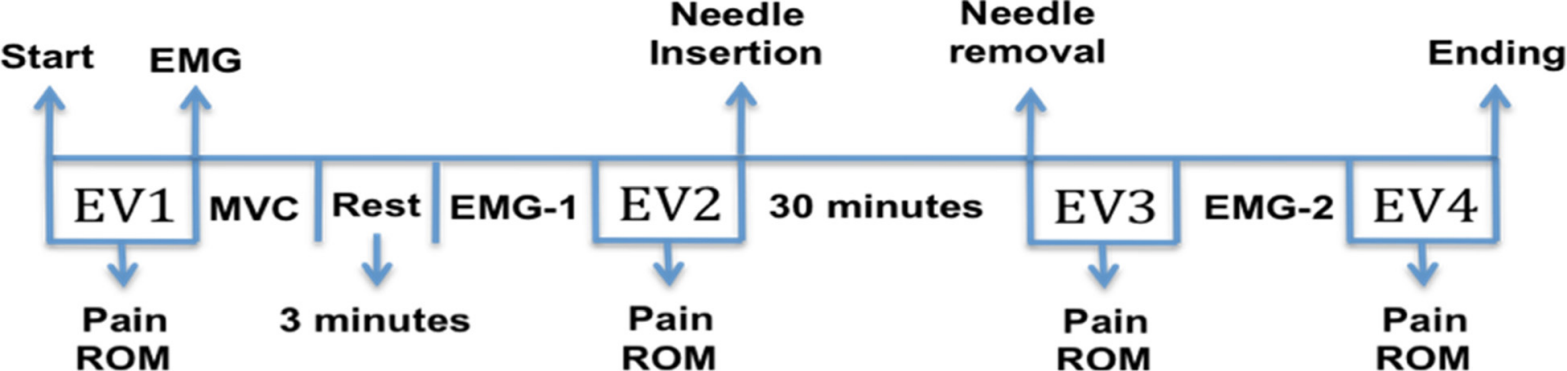
**Flow sequence diagram of data recording.** (EV1, pain and cervical range of motion evaluation; EV2, Second pain and cervical range of motion evaluation, EV3, Third pain and cervical range of motion evaluation; EV4, Fourth pain and cervical range of motion evaluation; EMG-1, First electromyographic evaluation ; EMG-2, Second electromyographic evaluation; ROM, Range of motion; EMG, Electromyography; MVC, Maximal voluntary contraction).

Figure 2 displays the sequence of the experiment. The evaluations will be performed with the patient sitting comfortably in a chair with both feet flat on the floor, hips and knees at flexed at 90°, buttocks positioned against the back of the chair and treated shoulder unclothed. Data on pain (numerical rating scale and pain area) and cervical range of motion data will be collected at baseline (EV1).

**Figure 3:**
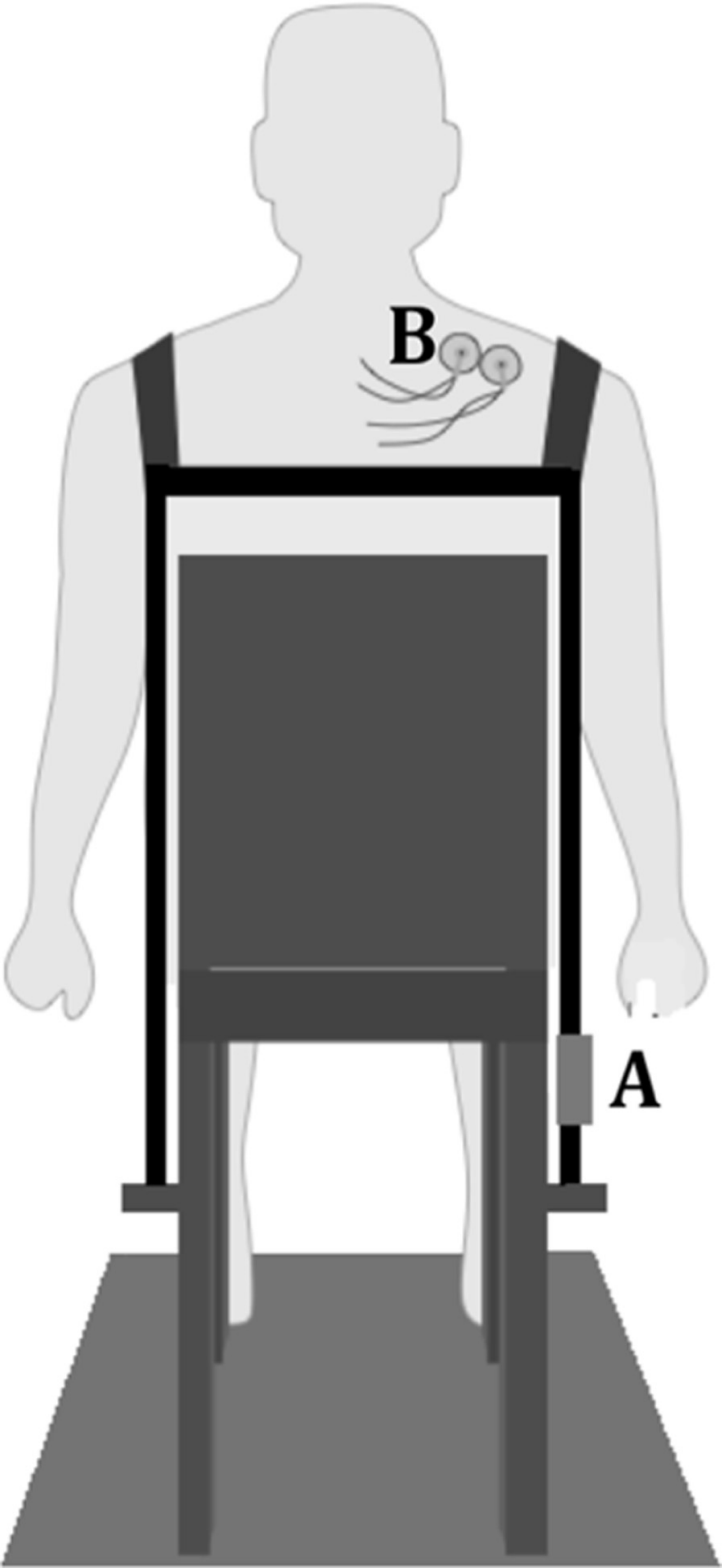
**Details of experimental setup.** Two straps over the acromion -one attached directly to the chair and one attached to the support of a load cell **(A)**; Force and electromyography activity (**(B)** surface electrode) of the upper trapezius muscle will be measured simultaneously during shoulder elevation movements. Feedback will be provided from the projection of a 20-inch screen on a white wall in front of the subject.

Disposable electrodes will be attached for the collection of the sEMG signal in the upper trapezius muscle treated with acupuncture. Straps will be hung from the shoulders and connected in front and back by another strap with Velcro® to allow adjustments for the chest size of each individual. The ipsilateral strap to the shoulder on which the sEMG signal will be read will be attached to a load cell (EMG System do Brazil Ltda, São José dos Campos, São Paulo, Brazil) connected to a support attached to the chair (Figure 3). The strap on the contralateral shoulder will be attached directly to the chair. The straps will be individually adjusted and the volunteer will be instructed to raise the shoulder to be analyzed in maximum voluntary contraction (MVC) for five seconds, with a three-minute rest interval between readings.

The maximum value in Newtons (N) will be considered the MVC. After three minutes of rest following the last MVC, the first sEMG signal (EMG-1) will be collected. The volunteer will be instructed to perform a ‘step contraction’, consisting of five force levels (10, 15, 20, 25 and 30% MVC). To investigate possible myoelectric manifestations of fatigue, a repetition of the third step (20% MVC) will be added as the sixth step to determine differences in the sEMG variables between these two steps [22]. Contraction time will be 11 seconds for each force level. Feedback of the step contraction will be provided from the projection of a 20-inch screen on a white wall in front of the subject. All participants will receive training prior to the shoulder elevations, based on the previously determined force levels.

New evaluations of pain and cervical range of motion (EV2) will be performed after one minute of rest. Acupuncture will then be performed, with the needles remaining inserted for 30 minutes. After the removal of the needles, further evaluations of pain and cervical range of motion will be performed (EV3), followed by a second EMG reading (EMG-2) in the same manner as performed during EMG-1. A fourth evaluation of pain and cervical range of motion (EV4) will be performed after two minutes of rest.

### Electromyography signal processing

For the analysis of the sEMG signal, the first second of each step (considered as a transition time between force levels) will be discarded and the subsequent 10 seconds will be divided into one-second windows. The root mean square (RMS) and median frequency (MDF) of the power spectrum will be calculated for each window. All EMG signals will be processed performing specific routines carried out in the Matlab program, version 7.1 (MathWorks Inc., Natick, Massachusetts, United States).

### Sample size calculation

The sample size was calculated considering α = 0.05 (5% chance of type one error) and 1-β = 0.99 (% of power of the sample), and data on the amplitude of the EMG signal in the study by Chou *et al*. [26]. The values used were those described by Chou et al. [26] in the period prior to acupuncture (21.3 ± 9.5 μV) and after three minutes of needle manipulation in the acupoints TE-5 and LI-11 (9.5 ± 3.5 μV). The minimum number for each group was determined to be 12 individuals (total = 24).

### Data analysis

Linear regression analysis will be applied for each individual to investigate associations between the sEMG variables and force (excluding the sixth step). The slopes of the regression lines will be used to measure the sensitivity of the sEMG variables regarding changes in force. The Shapiro-Wilk test will be used to test the normality of the data distribution regarding RMS and MDF values in the third and sixth step of the step contraction. Paired samples (Student’s t-test or Wilcoxon test) will be used to test differences between third and sixth contraction step. A mixed linear model will be used to analyze and compare the slopes of the regression lines during sustained contraction (five steps) between pre-acupuncture (EMG-1) and post-acupuncture (EMG-2) evaluations. These comparisons will demonstrate whether acupoints TE-5 and LI-11 affect the activity of the upper trapezius muscle, as hypothesized. The pain (numerical rating scale and pain area) and cervical range of motion data collected during the four evaluations will also be compared using a mixed linear model. The Statistical Package for Social Sciences (**IBM SPSS Statistics**, version 20.0, IBM™, Armonk, New York, United States) for Windows) will be employed for the statistical analysis, with the level of significance set to 5% (*P* <0.05).

### Ethical and data security

This trial received approval from the Human Research Ethics Committee of University Nove de Julho (Brazil) under process number 525.849, dated 12 March 2012. All individuals will be asked to provide written informed consent prior to randomization using a standard form. This trail was registered with the World Health Organization at Clinicaltrials.gov under number NCT01984021 on 7 November 2013.

## Discussion

The purpose of this randomized clinical trial is to evaluate the immediate effect of acupuncture on pain, cervical range of motion and EMG activity of the upper trapezius muscle in patients with NS-NP. The study will support the practice of evidence-based physical therapy for individuals with NS-NP. Data will be published after the study is completed.

## Trial status

Patient recruitment is currently underway. Started on March 2015 and expected to finish on June 2015.

## Abbreviations

CG: Control group
EMG: Electromyography
LI-11: Large intestine 11
MDF: Median frequency
MVC: Maximum voluntary contraction
NPG: Neck pain group
NS-NP: Nonspecific neck pain
RMS: Root mean square
sACP: Sham acupuncture
sEMG: Surface electromyography
tACP: Traditional acupuncture
TE-5: Triple energizer 5
MVC: Maximal voluntary contraction
